# Everybody's Page

**Published:** 1890-01-04

**Authors:** 


### Everybody's Page.

Vinegar and water is the best application for fleabites.

Ocular headaches often cause dizziness, but catarrhal neuralgia seldom, if ever, does so.

The existence of numerous small municipalities in Australia, which can only pay the necessary salaries and current expenses, is found to be a great bar to sanitary advancement.

The proportion of deaths per thousand in the metropolitan area of Melbourne is double that in the country. This is not surprising when we are told that the condition of West Melbourne swamp is so bad that it is a danger to the health of the community, and probably the cause of much zymotic disease.

A simple way of detecting the existence of defects in bath-wastes and untrapped sinks is to pour peppermint, or some other liquid with a pungent smell, down a gulley outside the house. All the windows must first be closed. The presence of this pungent smell at any of the suspected points will be sure evidence that something is wrong.

A quack once told Abernethy that the reason he gained so many clients was that so very few out of every hundred people were capable of appreciating cultivation in others. "The few," said the quack, "go to you, the rest come to me." Not only is the number of intelligent people who appreciate cultivation small, but many who can and should do so are from sheer laziness only too ready to put up with an inferior article.

"Anything will do so long as I have no trouble" might be the motto of the modern disciple of Lao Tzŭ, the Chinese moralist and apostle of Tao, who taught that all the evils of life come from action. But such a doctrine as this cannot find many followers, except amongst opium eaters and vacuous dreamers, in an age the very essence of which is activity. Those who have to seek mental refuge from misery—and they are many—seek it in these practical days in action rather than in the idealising of inaction.

There is no doubt that in England most people eat too much animal food and far too few vegetables, and we might vary our daily *menu* very easily and pleasantly to the benefit of our health. We nearly all boil our vegetable marrows and put melted butter on them, but they are excellent stewed in thick gravy or stuffed and baked, and tomatoes cooked in the same way are equally good. Tiny mutton cutlets smothered in a variety of vegetables can be had in summer and winter; cauliflower and tomatoes *au gratin*; rice stewed in good gravy with tomatoes cut up in it. These are a few among the many dishes that a good cook could think of and which would help to lessen the quantity of animal food.

In spite of all that has been done and all that is being done to reduce the rents paid by poor people and to increase their comfort, central London seems as though it must always prove a difficulty, for the reason that there is an enormous population which must of necessity live close to the centre of their work. For instance, the costers must be near the markets or in St. Pancras, where they do such a good trade; the artificial flower workers know very well that if they want to get work they must be on the spot, and the dock labourers must always be on the look out for jobs that may appear at any moment. And so all these poor folk crowd into certain quarters of the city, and this of necessity raises the rents to the exorbitant prices we so often hear of.

The protection of the spine is almost more efficacious as a preventive of sunstroke than the protection of the head.

Twenty anti-rabic institutes have been founded in different parts of the world since Pasteur's discovery in 1885.

Oil of eucalyptus is asserted to be an excellent disguiser of the taste of cod liver oil. But does not the eucalyptus oil in its turn also need a disguiser?

A new sort of compressed bread for the use of troops has been invented, which is made of ordinary flour and is in the form of thin cakes. When wanted for use the bread is soaked for a minute in water and then exposed to the air for a quarter of an hour, when it resumes its original size and tastes exactly like fresh bread.

The recent disaster in an American theatre brings home to us unpleasantly the not very remote possibility of similar catastrophes in this country. Managers here might with advantage take a hint from the health authorities of the large cities in the States, who require plans of theatres showing the exits to be printed on the backs of the programmes.

It is stated that the unpleasant smell which not unfrequently makes a refrigerator disagreeable can, if the smell arises from dampness, be remedied and prevented by placing some fresh quicklime on a plate inside the refrigerator. The lime, which absorbs one-third its weight of moisture, not only dries the air, but increases in no small measure the effect of the coolness of the ice.

Inasmuch as a good garter which combines the maximum of support with the minimum of constriction has yet to be invented, the sooner it is discarded for the more sensible suspenders, the better for health. It is practically impossible to get a garter which does not impede the flow of venous blood, and even if varicose veins occur oftener in men than in women, this fact would not be proof that the garter is no source of disease.

The prevalence of typhoid fever and diphtheria in Melbourne has led Dr. Jamieson, the health officer of the city, to insist once more on the necessity of adopting a comprehensive system of underground drainage. Experiments for deodorising street sewage have been carried on by him and the city surveyor, and the substances tried are sulphate of iron, Süvren's disinfectant, lime, manganate of soda, and sanitas. They report most favourably of sulphate of iron as being effective, comparatively cheap and convenient for application, and free from smell, but neither it nor any of the other agents can be depended on to destroy completely the offensive smell arising from drains unless used in very large quantities.

As a proof of atmospheric agency being one of the causes of influenza, the late Sir Thomas Watson stated in his lectures that on April 3, 1833, London was smitten with the disease, and on the same day the "Stag" frigate was coming up Channel, and arrived at two o'clock off Berry Head, Devon, all hands at that time being well on board. Half an hour afterwards, the wind being easterly, and blowing off the land, forty men were down with influenza, by six o'clock the number was increased to sixty, and by two o'clock the next day to 160. On the self-same evening a regiment stationed at Portsmouth was in a perfectly healthy state, but by the next morning so many of the men were afflicted with influenza that the garrison duty could not be performed.

